# Progress in Surgery

**Published:** 1899-01-21

**Authors:** 


### Progress in Surgery.

### DISEASES OF THE PHARYNX.

**Pneumococci.**—The secretion from the tonsils of forty healthy persons of all ages, living under the most diverse conditions, has been examined bacteriologically by Bezancon and Griffon,[1] and reported to the Société des Hôpitaux, Paris. They used the serum of a young rabbit as a culture medium, and they found pneumococci present in every individual. They believe that imperfect methods of investigation must have been pursued in previous observations in which pneumococci were found in a much lower proportion of healthy throats.

**Tonsillitis.**—A case of croupous tonsillitis of staphylococcus origin with fatal perforation of the internal carotid artery is reported by Jacobi and Ewing.[2] There were no Klebs-Löffler bacilli, and in two weeks from the onset of the attack the throat was pronounced clear and the child much better. Then came a rigor and rise of temperature, pain in the throat, swelling of glands in the neck, and two days later severe hæmorrhage from the nose. The hæmorrhage recurred once or twice before the last fatal hæmorrhage occurred. The autopsy revealed the fatal hæmorrhage from an ulcerous perforation of the right internal carotid artery and the pharyngeal wall. References are given to some similar cases of pharyngeal hæmorrhage.

**Tonsils, Chronic Enlargement of.**—Hugh Walsham draws attention to the existence of cartilage and bone in the faucial tonsils. He considers that they are vestigial in origin. Kanthack believes that they are due to some metaplastic process. Wyatt Wingrave[4] considers them to be developmental vestiges, identical with the subpharyngeal cartilage of Luschka. Wingrave's experience is that cartilaginous nodules are by no means rare in this situation. He says that it is not unusual to find considerable resistance in tonsillotomy disproportionate to the density of the tonsil tissue, and that he has in several instances—all in young subjects—proved this to be due to the presence of fibrous and cartilaginous tissue.

**Latent Tuberculosis of the Tonsil.**—Walsham's[5] observations on cartilage in the tonsils occurred in the course of his practically much more important investigation into the question of tuberculous infection of the glands of the neck through the tonsils, an opportunity for which he had in his capacity as pathologist to the City of London Hospital for Diseases of the Chest. He examined histologically the tonsils and the follicular glands at the base of the tongue of every case of tuberculosis coming before him for post-mortem examination, and also further examined in the same way portions of tonsils and adenoids removed in the throat department at Bartholomew's Hospital, which Mr. Bowlby submitted to him for the purpose. Walsham thinks that the results of his investigations warrant the following conclusions: That the tonsils are very frequently infected, and that tubercle may be primary in the tonsil. They are frequently affected secondarily in chronic pulmonary tuberculosis, and that then the cervical glands are frequently affected secondarily from the tonsils; but that the follicles at the base of the tongue are rarely tuberculous. Lastly, that the tonsil may be affected from without or through the blood stream in acute miliary tuberculosis.

**Septic Inflammation.**—Two cases of acute diffuse cellulitis of the submaxillary region (angina ludovici) in which tracheotomy was performed are recorded by Webber.[6] In the successful case the whole of the tissues between the chin and the sternum, and between the sterno-mastoid muscles on each side, were then in a condition of brawny, inflammatory œdema. Only three days before she was well, when she had fallen against the sharp edge of a chair and had bruised or cut her chin. Dyspnœa became urgent, and an incision was made deeply from chin to sternum, yielding dark blood and serum; it was rapidly deepened, until at about five inches from the surface of the skin the trachea was reached. The next day but one she was ordered two grains of quinine every four hours, and a pint of champagne and four ounces of brandy in the 24 hours.

**Adenoids.**—Now that the operation for adenoid vegetations is very frequently performed under the impression that the procedure is very simple and altogether safe, it is well to listen to warning notes that every now and then are uttered by those whose experience leads to the conclusion that considerable care and skill are called for if the dangerous factors are to be excluded and the operation efficiently carried out. The profession entertains a sense of gratitude to those operators who report their misfortunes for the benefit of others, and it is with this feeling we direct attention to a discussion opened before the American Laryngological Association by a skilled laryngologist, F. W. Hinkel,[7] wherein he reports a death following immediately an operation for naso-pharyngeal adenoids under chloroform. The patient was a boy aged six years, with recurring earache and deafness for four years. He had occasional attacks of hacking cough, but was not a mouth breather or snorer, and had not the usual expression or voice of a sufferer from adenoids. There was a moderate amount of lymphoid tissue in the vault of the pharynx. The chloroform was given by an experienced anæsthetist, who was familiar with the difficulties attending the administration of an anæsthetic in these cases. He was accustomed to apply the mask dry and then administer the chloroform a few drops at a time; in this way a very small amount usually sufficed. There was in this patient a very slight cardiac systolic murmur, loudest at the base, with the apex beat in normal position. This, in view of the normal size and position of the heart, he regarded as probably not due to organic leison, its presence being common in debilitated children. Vomiting of undigested food taken five hours previously delayed the operation. Respiration was interrupted a number of times by spasm of the glottis. During the operation, which required a renewed administration of chloroform, careful watch was kept on the pulse and respiration, and on removal of the chloroform to make way for the operation to proceed, he noted the temporal pulse. It was good. The operation was completed, when suddenly the boy gave a few hurried shallow gasps and respiration ceased. The pulse had disappeared, and no cardiac impulse could be felt or heard, the pupils were dilated. Hypodermics of strychnine and digitalis which lay prepared were immediately injected; the patient was inverted, and artificial respiration begun. Further injections of ether, atropine, digitalis, and strychnine were given; hot water injections and cold affusions were used, but all in vain. No post-mortem was obtained. Death here occurred without warning, after the chloroform had been removed two or three minutes, with almost simultaneous failure of pulse and respiration. Hinkel thinks death resulted from central nervous disturbances, as in the class of chloroform deaths termed by Lauder Brunton[8] "neuro-paralysis," in which not only respiration and circulation, but all the functions of the nervous system fail together; the breathing stops, the pulse stops, and the person dies instantaneously and quite quietly without convulsions. Hinkel remarks that "we have all observed the frequency with which spasm of the glottis and temporary cessation of respiration occur in the administration of an anæsthetic for this operation," and that Lauder Brunton points out that spasm of the glottis is occasionally a cause of death in chloroform narcosis. Hinkel directs attention to other communications on the alarming mortality in the adenoid operation and tonsillotomy under chloroform. Thus in 1896 Holloway[9] tabulated fourteen deaths under chloroform in nose and throat operations reported in England up to April, 1895. Of these, eleven were in operations on the tonsils and naso-pharyngeal adenoids reported since 1892. In addition to these eleven cases, Hinkel has been able to collect nine others, including his own case, making eighteen in all. Six of the operations were for adenoids alone, three for adenoids and hypertrophied tonsils, two for hypertrophied tonsils alone. In four cases death occurred before the operation was begun; in three from a few minutes to an hour after the operation was completed.

Hinkel argues that as the deaths from chloroform in operations of all kinds have been placed at about 1 in 4,000, this death-roll of eighteen would imply that 72,000 of these operations had been performed under chloroform since 1892, a preposterous conclusion, unless there be a special susceptibility in the subjects of adenoids. Moreover, it must be remembered that most of the reported deaths have occurred in England alone, and that we may infer that all deaths under chloroform in this operation have not been reported.

Hinkel cites the observations of the Vienna pathologists, Paltauf[10] and Kolisko,[11] viz., that it has been found post-mortem in a number of cases of sudden death from slight causes that there was present hypertrophy of the lymphoid tissue throughout the body, including the tonsils, the lymphoid structures at the root of the tongue, and the naso-pharyngeal adenoids. The thymus gland was persistent and often very large, and the intestinal follicles were markedly hypertrophied. In addition there was frequently present a dilated heart not dependent on valvular lesions, and at times a narrowing of the aorta with small size of the peripheral vessels. This condition, which has been called the *habitus lymphaticus*, was found, among others, in a number of cases of death during chloroform administration. Picqué[12] has cited a number of cases of sudden death in young children in which this enlargement of the thymus and other lymphoid structures was present, death in all being very sudden.

Paltauf states that the exaggerated development of the thymus or its abnormal persistence constitutes a concomitant symptom, as does also the hypertrophy of the lymphatic ganglions or tonsils, and that as a result there is an increased vulnerability of the individual and a particular predisposition to cardiac syncope. That is, these untoward factors are found in the constitutional condition, one of whose manifestations is the hypertrophied naso-pharyngeal lymphoid tissue for which we so frequently operate.

We may here recall incidentally two recently recorded cases of fatal dyspnœa from hypertrophied thyroid gland. One is reported by Barnett[13] of a male child which soon after birth developed all the symptoms of laryngeal obstruction. Tracheotomy had improved the condition for several weeks, when the child died during a prolonged attack of dyspnœa. The autopsy showed the larynx and trachea to be normal and the thymus enlarged. It was thought possible that as the thymus had outlying lobules in the neck they may have caused pressure upon the recurrent laryngeal nerves. The other case is recorded by Clessin. A healthy child, two months old, was found dead in bed one morning, though at ten o'clock the night before it had appeared healthy. At the autopsy all the organs were normal, except that the thymus, which was much enlarged, so compressing the trachea that a pin could scarcely be passed into the tube, and that the lungs were congested, and they and the heart showed petechial hæmorrhages. It is supposed that death was due to sudden swelling of the thymus, as it was very congested. Clessin failed to produce death in pups by tying the thymus veins.

Hinkel can only find six cases recorded of death attributable to the adenoid operation, all from hæmorrhage, usually secondary, and in the greater number occurring in "bleeders," viz.: (1) Hooper, Trans. Amer. Lar. Ass., 1889, p. 56; (2) Delavan, ditto, 1892, p. 8; (3) Newcourt, Amer. Jour. of Med. Sc., Nov., 1893; (4) Barkan, Occid. Med. Times, Vol. VIII., 1894; (5) Ichmiegelow, Monat. f. Ohrenheil, Vol. XXXI, 1897; (6) Kenefick's case, Laryngoscope, April, 1898. Collected by Newcourt.

In conclusion, Hinkel condemns entirely the use of chloroform in these cases, except under some peculiar circumstances, and advises the employment of ethyl bromide, nitrous oxide, or ether.

In the discussion following, Leland mentioned another fatal case from hæmorrhage in a child age 11, who had apparently recovered from the usual adenoid operation, when, on the seventh day, while the child was walking out of doors, copious hæmorrhage came on, and recurred once or twice, the last attack on the next day being fatal, while Hopkins spoke of a case in his own experience where the child, who had been operated upon under chloroform, rallied, though for an interval the child was thought to be dead.

Another case of fatal secondary hæmorrhage after removal of adenoids is reported by Preble.[14] It occurred on the eighth day after operation.

Bryson Delavan relies entirely upon ether now for adenoid cases; he has never known secondary evils, such as bronchitis or pneumonia, to arise, and he does not believe that it causes any marked increase in the bleeding. Hopkins also employs ether, while Wagner-Gleitsmann, and Hinkel use by preference ethyl-bromide.

Hinkel states that Roaldes had written to him saying that he had used bromide of ethyl 2,500 times with satisfaction and without accident, while Gougenheim, Luc, and Moure had also written that they had used it in tonsil and adenoid operations many hundreds of times with safety and satisfaction. Hinkel also uses nitrous oxide, while Swain, Logan, and Thrasher still prefer chloroform.

**Stammerers.**—Makuen referred to several cases of stammerers, one of whom never stammered after removal of adenoids, while others were much improved. Delavan supported the statement that the removal of lymphoid hypertrophies has a marked effect in the relief of certain defects of speech, due not only to restoration of the normal space of the pharynx as a resonating cavity, but still more to the relief of catarrhal conditions of the general relaxation of the parts which accompany them.

[1] Phil. Med. Journ., 1898, No. 21; Amer. Journ. of Med. Sci., Sept., 1898. [2] Phil. Med. Journ., 1898, No. 23; Amer. Journ. Med. Sci., Sept., 1898. [3] Lancet, Aug. 13, 1898. [4] Lancet, July 17, 1898. [5] Lancet, June 18, 1898. [6] Lancet, Sept. 17, 1898. [7] New York Med. Journ., Oct. 29, 1898. [8] Lancet, July 6, 1895. [9] Med. Mag. London, vol. v., p. 598. [10] Med. News, Aug. 7, 1897. [11] Foster's Therap., vol. i., p. 398. [12] Bull. et Mem. de la Soc. Chir. Paris, xxi., 1895. [13] Lancet, April 30, 1898. [14] Boston Med. and Surg. Jour., May 19, 1898; Amer. Journ. of Med. Sci., Sept., 1898.

